# Impact of mutant *β*-catenin on ABCB1 expression and therapy response in colon cancer cells

**DOI:** 10.1038/bjc.2012.81

**Published:** 2012-03-29

**Authors:** U Stein, C Fleuter, F Siegel, J Smith, A Kopacek, D A Scudiero, K M Hite, P M Schlag, R H Shoemaker, W Walther

**Affiliations:** 1 Experimental and Clinical Research Center, a joint cooperation between the Charité Medical Faculty and the Max-Delbrück-Center for Molecular Medicine, Berlin 13125, Germany; 2 SAIC-Frederick, National Cancer Institute-Frederick, Frederick, MD 21702, USA; 3 Charité Comprehensive Cancer Center, Charité University Medicine Berlin, Berlin 10117, Germany; 4 Screening Technologies Branch, Developmental Therapeutics Program, Division of Cancer Treatment and Diagnosis, National Cancer Institute-Frederick, Frederick, MD 21702, USA

**Keywords:** colon cancer, *β*-catenin, multidrug resistance, ABCB1, therapy response

## Abstract

**Background:**

Colorectal cancers are often chemoresistant toward antitumour drugs that are substrates for ABCB1-mediated multidrug resistance (MDR). Activation of the Wnt/*β*-catenin pathway is frequently observed in colorectal cancers. This study investigates the impact of activated, gain-of-function *β*-catenin on the chemoresistant phenotype.

**Methods:**

The effect of mutant (mut) *β*-catenin on ABCB1 expression and promoter activity was examined using HCT116 human colon cancer cells and isogenic sublines harbouring gain-of-function or wild-type *β*-catenin, and patients’ tumours. Chemosensitivity towards 24 anticancer drugs was determined by high throughput screening.

**Results:**

Cell lines with mut *β*-catenin showed high ABCB1 promoter activity and expression. Transfection and siRNA studies demonstrated a dominant role for the mutant allele in activating ABCB1 expression. Patients’ primary colon cancer tumours shown to express the same mut *β*-catenin allele also expressed high ABCB1 levels. However, cell line chemosensitivities towards 24 MDR-related and non-related antitumour drugs did not differ despite different *β*-catenin genotypes.

**Conclusion:**

Although ABCB1 is dominantly regulated by mut *β*-catenin, this did not lead to drug resistance in the isogenic cell line model studied. In patient samples, the same *β*-catenin mutation was detected. The functional significance of the mutation for predicting patients’ therapy response or for individualisation of chemotherapy regimens remains to be established.

Over 90% of colorectal cancers bear mutations in the Wnt/*β*-catenin signalling pathway, notably APC and *β*-catenin, that result in the activation of this pathway (Fearon and Vogelstein, 1990; Bienz and Clevers, 2000; Polakis, 2000; Vogelstein and Kinzler, 2004; Klaus and Birchmeier, 2008; Najdi *et al*, 2011). Activating mutations of *β*-catenin affect either the assembly of the phosphorylation complex or the phosphorylation sites on *β*-catenin. The most frequently observed mutations in *β*-catenin involve the deletion or the exchange of serine and threonine residues at the positions 45, 41, 37 and 33; interfering with its efficient ubiquitination and degradation in the proteasome (Orford *et al*, 1997).

Such mutations are found in a wide variety of human cancers, including the colon, pancreatic, gastric, ovarian and prostate cancer as well as melanoma (Polakis, 2000). They are associated with aggressive tumour growth and poor prognosis, and accumulation of *β*-catenin in the nucleus has been correlated with late stages of tumour progression and the development of metastases (Ilyas *et al*, 1997; Morin *et al*, 1997; Polakis, 1999; Kim *et al*, 2003; Provost *et al*, 2003; Wong *et al*, 2004; Stein *et al*, 2006).

The phenomenon of multidrug resistance (MDR) was identified as one of the most frequent causes for therapy resistance in cancer and represents a major cause of failure of cancer chemotherapy (Gottesman *et al*, 2002; Stein and Walther, 2006; Szakacs *et al*, 2006; Tiwari *et al*, 2011). The development of MDR is mainly dependent on the expression of MDR-associated genes encoding ABC transporter proteins (Gillet and Gottesman, 2011). The MDR gene *ABCB1* (MDR1) encoding the gene product P-glycoprotein was the first human ABC transporter cloned. The generation of the MDR phenotype was shown directly by ABCB1 cDNA transfection (Riordan and Ling, 1979; Ueda *et al*, 1987). ABCB1 acts as a drug efflux pump lowering the intracellular concentration of cytotoxic drugs. It transports a wide spectrum of hydrophobic, neutral or positively charged substrates such as taxanes and anthracyclines (Tusnady *et al*, 2006; Tiwari *et al*, 2011).

High ABCB1 levels have been detected in normal tissues with excretory or secretory function, which include colorectal epithelium, and in tumours originating from these organs (Thiebaut *et al*, 1987; Cordon-Cardo *et al*, 1990). Overexpression of ABCB1 correlates with a negative prognosis in several types of cancer. ABCB1 expression is inherently overexpressed in tumours of the colon, making them primarily chemotherapy-resistant towards a wide panel of anticancer drugs. Consequently, there is only a limited selection of chemotherapeutics for treatment of gastrointestinal cancer (Weinstein *et al*, 1991; Litman *et al*, 2001; Ho *et al*, 2003; Tiwari *et al*, 2011).

Reports of T-cell factor 4 (TCF4)-binding sites in the *ABCB1* gene promoter (Yamada *et al*, 2000, 2003) suggest that *β*-catenin/TCF4 signalling could provide an underlying mechanism contributing to the chemoresistance phenotype. In this report, we have utilised isogenic colon cancer cell lines to investigate the effects of a common oncogenic *β*-catenin mutation on chemoresistance under conditions where the tumour genotype could be controlled experimentally and sought to confirm the findings using patient tumour samples. We used high throughput screening with MDR-related and non-related antitumour compounds in order to evaluate the impact of mutant (mut) *β*-catenin on *in vitro* drug sensitivity.

## Materials and methods

### Tumour cell lines, transfections and sulindac treatment

The human colon carcinoma cell line HCT116 showed a moderate ranking in terms of MDR amongst the 60 cell line panel of the National Cancer Institute (Wu *et al*, 1992; Izquierdo *et al*, 1996), making these cells and isogenic derivatives suitable models for investigating modulation of chemosensitivity. HCT116 cells (heterozygous for *β*-catenin Δ45, exon 3; resulting in the loss of the serine phosphorylation site, S45), the *β*-catenin knockout cell lines HAB-18^mut^ and HAB-68^mut^ (express only the mut *β*-catenin allele), and HAB-85^wt^ and HAB-92^wt^ (express only one wild-type (wt) *β*-catenin allele) were kindly obtained from Todd Waldman, Lombardi Comprehensive Cancer Center, Georgetown University School of Medicine, Washington, DC (Kim *et al*, 2002). *β*-Catenin genotypes were previously confirmed by sequencing exon 3 and by reverse transcription (RT)–PCR-based restriction fragment length polymorphism (Stein *et al*, 2006). Transfections of wt and Δ45-mut *β*-catenin cDNA, kindly provided by Bert Vogelstein, Johns Hopkins University, Baltimore MD, USA, were performed using lipofectin. *β*-Catenin siRNA (*β*-catenin_1 sense 5′-GGUGGUGGUUAAUAAGGCU-3′, *β*-catenin_1 antisense 5′-GCCUUAUUAACCACCACC-3′, *β*-catenin_2 sense 5′-CCUAUACUUACGAAAAACU-3′, *β*-catenin_2 antisense 5′-AGUUUUUCGUAAGUAUAGG-3′) or scrambled control siRNA (all from Ambion, Austin, TX, USA) were transfected using oligofectamine. For each transfection experiment, at least three independent transfected clones of each cell line were analysed; representative clones are shown. Sulindac treatment was performed with 100 *μ*M sulindac sulphide (Sigma, Munich, Germany; Boon *et al*, 2004) dissolved in dimethyl sulfoxide and diluted in growth medium for 24 h.

### Patients and tumour tissues

Tissue specimens from 33 colon cancer patients (20 male, 13 female; age range 54–93) were obtained with written consent of the patients (approved by the local ethics committee of the Charité, Berlin). These patients with adenocarcinomas of UICC stages I–III were previously untreated, did not have a history of familial colon cancer, did not suffer from a second tumour of the same or a different entity, underwent surgical R0 resection, and had not developed distant metastases at time of surgery. Tumour specimens were snap-frozen in liquid nitrogen and blinded for analysis. Three of these patients were identified to harbour the heterozygous in-frame deletion mutation of Δ45 in exon 3 of the *β*-catenin gene (Stein *et al*, 2006). In order to analyse a potential correlation of mut *β*-catenin with its nuclear localisation and ABCB1 expression levels, serial cryosections were made for immunohistochemistry.

### *ABCB1* gene promoter analysis

The influence of *β*-catenin/TCF4-mediated gene expression was analysed in pTOP-CAT- and pFOP-CAT- (kindly provided by Walter Birchmeier, Max-Delbrück-Center for Molecular Medicine, Berlin, Germany) transfected cells. pTOP-CAT utilises the multimerised TCF4 consensus DNA-binding sequence to drive the CAT-reporter expression (van de Wetering *et al*, 2002). pFOP-CAT contains a mutated TCF4 sequence and serves to establish the level of background, non-specific signal. The ABCB1 promoter CAT-construct pMDR-CAT1 (−1974 to +121) was kindly provided by Kimitoshi Kohno, University of Occupational and Environmental Health, Kitakyushu, Japan (Kohno *et al*, 1990). For CAT-ELISA, the promoter-less plasmid pCAT-Basic (Promega, Mannheim, Germany) and transfections without DNA served as controls. Transfer efficiency was controlled by transfection of the GFP-expressing pEGFP-N1 plasmid (Clontech, Heidelberg, Germany) and subsequent flow cytometry. Transfections and CAT-ELISA were carried out as described previously (Stein *et al*, 2006). The amount of CAT protein was normalised to the protein content of the respective lysate, expressed as pg CAT per mg protein, and calculated as percentage of CAT reporter gene expression in HCT116 cells. Values are given as average of triplicates.

### Quantitative real-time RT–PCR

RT reaction was performed with 100 ng of total RNA (MuLV Reverse Transcriptase, Applied Biosystems, Weiterstadt, Germany). Quantitative real-time PCR (95 °C 60 s, 45 cycles of 95 °C 10 s, 62 °C 10 s, 72 °C 20 s) was performed using the LightCycler (DNA Master Hybridization Probes Kit, Roche Diagnostics, Mannheim, Germany) as previously described (Stein *et al*, 2002). Expression of *ABCB1*, *ABCC1*, *ABCG2*, *MVP* and of the housekeeping gene glucose-6-phosphate dehydrogenase (*G6PDH*; h-G6PDH Housekeeping Gene Set, Roche) was determined in parallel from the same RT reaction by using gene-specific hybridisation probes, each done in duplicate per sample. For ABCB1: forward primer 5′-TTGAAATGAAAATGTTGTCTGG-3′, reverse primer 5′-CAAAGAAACAACGGTTCGG-3′, FITC probe 5′-CACTGAAAGATAAGAAAGAACTAGAAGGTGCT-3′, LCRed640 probe 5′-GGAAGATCGCTACTGAAGCAATAGAAAACT-3′ for ABCC1: forward primer 5′-TGCCGAAGGAGAGATCATCATC-3′, reverse primer 5′-CGGAGGGAACCCGAAAACA-3′ FITC probe 5′-GCCTGCACGACCTCCGCTTCAAGA-3′, LCRed640 probe 5′-CACCATCATCCCCCAGGACCCTGTT-3′ for ABCG2: forward primer 5′-AGCAGGGACGAACAATCATC-3′, reverse primer 5′-AGGCCCGTGGAACATAAGTC-3′ FITC probe 5′-TCATCAGCCTCGATATTCCATCTTCAAGTT-3′, LCRed640 probe 5′-TTTGATAGCCTCACCTTATTGGCCTCAG-3′ for MVP: forward primer 5′-CGCATCCCCCCATACCACTA-3′, reverse primer 5′-GGCAAACAGTACCCTCTCATTGTCC-3′ FITC probe 5′-CAGAACAGCAACGTGTCCCGTGTGGA-3′, LCRed640 probe 5′-GTCGGGCCAAAGACCTACATCCGGC-3′ (syntheses of primers and probes: BioTeZ and TIB MolBiol, both Berlin, Germany). The calibrator cDNA was employed in serial dilutions simultaneously in each run, derived from the ABCB1-overexpressing cell line KBV-1 and the ABCC1-overexpressing cell line MCF-7/VP16 (kindly provided by Michael M Gottesman and Erasmus Schneider, National Cancer Institute, Bethesda, MD, USA), from the ABCG2-overexpressing cell line SW1573/2R120 and from the MVP-overexpressing cell line GLC-4/ADR (kindly provided by Henk J Broxterman, Free University Amsterdam, The Netherlands).

### Immunocytochemistry and immunohistochemistry

We used a monoclonal anti-ABCB1 antibody (Kamiya Biomedical, Seattle, WA, USA; 1 : 20) and a monoclonal anti-*β*-catenin antibody (BD Biosciences, Heidelberg, Germany, 1 : 800). For immunocytochemistry, cells were cultured on eight-well chamber slides; cells without primary antibody served as controls. For immunohistochemistry, consecutive cryosections were incubated on the same slide; tissue sections without primary antibodies served as controls. Cells or tissue sections were fixed, endogenous peroxidase was inactivated and cell membranes were permeabilised. After blocking, cells or tissue sections were incubated with the anti-ABCB1 or the anti-*β*-catenin antibody for 2 h. Detections were performed with the StreptABComplex/HRP Duet system (DAKO, Glostrup, Denmark). Nuclei were counterstained with hemalum (hematoxylin/alum mixture; Carl Roth GmbH, Karlsruhe, Germany). Microphotographs were taken with a Zeiss Axioplan 2 microscope and an Axiocam HRc camera (Zeiss, Göttingen, Germany) using the Axiovision 4.2 software (Zeiss).

### Immuno flow cytometry

We used a monoclonal anti-ABCB1 antibody (MRK16, antibodies-online, Atlanta, GA, USA; 1 : 20), a monoclonal anti-ABCC1 antibody (MRPm6, Acris antibodies, Herford, Germany; 1 : 20), a monoclonal anti-ABCG2 antibody (BXP-21, Abnova, Taipei, Taiwan; 1 : 20), a monoclonal anti-MVP antibody (MVP 1014, Gene Tex, Irvine, CA, USA; 1 : 20). Goat anti-mouse IgG1 and IgG2a antibodies were from Life Technologies (Darmstadt, Germany). Cells were prepared and were incubated as previously described (Stein *et al*, 1996). Fluorescence intensity of 10^4^ cells was measured with a FACSCalibur (Becton Dickinson, Franklin Lakes, NJ, USA) and expressed as mean fluorescence. After titration of the antibody, at least two independent experiments, each performed in duplicate, were carried out.

### Western blotting

Total protein extractions of the cells were performed with RIPA buffer (50 mM Tris-HCl (pH 7.5), 150 mM NaCl, 1% Nonidet P-40, supplemented with complete protease inhibitor tablets; Roche Diagnostics) for 30 min on ice. The nuclear, cytoplasmic and membrane protein fractions were isolated with the Qproteome Cell Compartment Kit (Qiagen, Hilden, Germany). Western blotting was carried out as described previously (Stein *et al*, 2009). Membranes were incubated overnight at 4 °C with a monoclonal anti-ABCB1 antibody (C219, Novus Biologicals, Cambridge, UK; 1 : 50), a monoclonal anti-ABCC1 antibody (MRPm6, Acris antibodies; 1 : 50), a monoclonal anti-ABCG2 antibody (BXP-21, Abnova; 1 : 50) and a monoclonal anti-MVP antibody (MVP 1014, Gene Tex; 1 : 50). Western blotting for *β*-tubulin (Becton Dickinson; 1 : 1000) served as loading control.

### Rhodamine assay

HCT116 cells and the *β*-catenin knockout cell lines HAB-68^mut^ and HAB-92^wt^ were incubated for 10 or 15 min at 37 °C with rhodamine 123 (0.75 mg ml^−1^; Sigma, Taufkirchen, Germany), and were then kept in rhodamine 123-free medium for another 60 min at 37 °C. Fluorescence intensity of 1 × 10^4^ cells per group was measured in duplicate per sample by using the FACSCalibur (Becton Dickinson, Cell Quest program).

### High throughput drug sensitivity phenotyping

A library of 24 prototype drugs (Table 1) including well known MDR substrates, small molecules not associated with MDR and drugs targeting kinases and other potentially relevant targets was assembled, dissolved in dimethyl sulfoxide, further diluted in isopropanol and distributed to wells of 384-well plates with a liquid handling robot in a randomised fashion such that each dilution was present in three replicate dilutions at random locations across the plates. The plates were then dried down in a SpeedVac, sealed and stored frozen at −20 °C. For use in assays, plates were thawed, compounds were re-solubilised in dimethyl sulfoxide and further diluted with growth medium (RPMI-1640 supplemented with 5% foetal bovine serum and 2 mM glutamine). Aliquots of diluted compounds were then transferred to 384-well plates containing tumour cells and the plates were incubated for 4 days in a 37 °C, humidified incubator with an atmosphere of 5% CO_2_. Viable cell numbers were then evaluated using an Alamar Blue assay. Briefly, cells were treated with Alamar Blue dissolved in serum-free RPMI-1640 and incubated for 4 h. Plates were then read on a Wallac Victor reader (PerkinElmer) at an excitation wavelength of 530 nm and emission wavelength of 590 nm. Values for triplicate wells at each concentration were then averaged and expressed as percent of vehicle (dimethyl sulfoxide) control. Concentration-response curves were generated and IC_50_ values extracted from the curves by linear interpolation. Response of cell lines across the drug library was compared in terms of concentration-response curves, derived IC_50_ values and patterns of sensitivity in relation to genotype.

### Statistical analysis

Levels of statistical significance were evaluated by using the *t*-test or the non-parametric two-sided Mann–Whitney rank sum test depending on whether the data passed or failed a normal distribution test.

## Results

### ABCB1 expression is dependent on *β*-catenin mutation status

**Figure 1:**
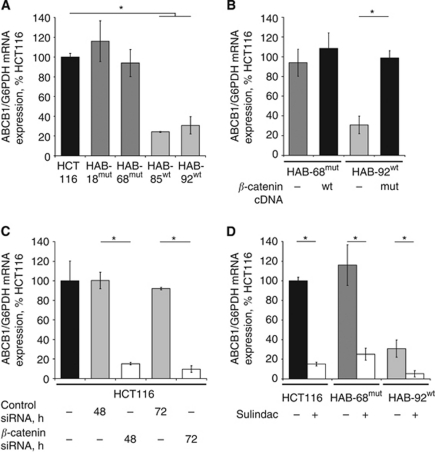
Gain-of-function *β*-catenin regulates ABCB1 mRNA expression in a dominant fashion. (**A**) ABCB1 mRNA expression analysis in HCT116, HAB-18^mut^, HAB-68^mut^, HAB-85^wt^ and HAB-92^wt^ cells. High ABCB1 levels were observed in the cell line HCT116 and its derivatives harbouring mut *β*-catenin, low ABCB1 expression in the derivatives with wt *β*-catenin. ABCB1 mRNA expression was determined by quantitative real-time RT–PCR; values represent the ratios of ABCB1 and G6PDH control mRNA (HCT116 cells: set 100%). Experiments were performed in duplicate and average values are shown. (**B**) ABCB1 mRNA expression in knockout derivatives with reconstituted *β*-catenin genotype. Transfection of HAB-68^mut^ cells with wt *β*-catenin cDNA did not change ABCB1 expression levels. Transfection of HAB-92^wt^ cells with mut *β*-catenin cDNA, however, induced ABCB1 expression up to four-fold. (**C**) ABCB1 mRNA expression in HCT116 cells following treatment with *β*-catenin siRNA. Knockdown of ABCB1 expression by *β*-catenin siRNA is shown. Transfection of control siRNA had no effect. (**D**) Treatment of HCT116, HAB-68^mut^ and HAB-92^wt^ cells with sulindac (100 *μ*M; 24 h). ABCB1 expression levels in solvent-treated cells were dependent on *β*-catenin genotype. Sulindac treatment reduced ABCB1 mRNA expression in HCT116, HAB-68^mut^ and HAB-92^wt^ cells. ^*^P<0.05.

Initially, we investigated the dependence of ABCB1 expression on the mutation status of *β*-catenin. We compared *ABCB1* gene expression levels in the parental human colon carcinoma cell line HCT116, that is heterozygous for an in-frame Δ45 deletion in exon 3 of the *β*-catenin gene, in the HAB-18^mut^ and HAB-68^mut^ as well as in the HAB-85^wt^ and HAB-92^wt^ knockout strains of HCT116 cells, in which either the wt or the mut *β*-catenin allele was ablated by homologous recombination (Kim *et al*, 2002). Derivatives that contain only the mut *β*-catenin allele showed high levels of ABCB1 mRNA, comparable to the parental cells, as determined by quantitative real-time RT–PCR. Derivatives that carry the wt *β*-catenin allele exclusively, showed up to four-fold lower ABCB1 expression levels (Figure 1A).

Next, we reconstituted the heterozygous *β*-catenin genotype of the knockout strains: HAB-68^mut^ cells were stably transfected with wt *β*-catenin cDNA and HAB-92^wt^ cells with Δ45-mutated *β*-catenin cDNA (empty vector transfections served as controls; Figure 1B). The reintroduction of the mut *β*-catenin allele into HAB-92^wt^ cells led to an up to four-fold increase in ABCB1 expression thereby restoring the expression level of the parental heterozygous HCT116 cell line. ABCB1 levels remained almost unchanged in HAB-68^mut^ cells following transfection of wt *β*-catenin. We also analysed *β*-catenin-controlled expression regulation of ABCB1 by treating HCT116 cells with siRNA acting on *β*-catenin (Figure 1C). A clear, up to 10-fold reduction in ABCB1 expression was measured 48 h and 72 h post siRNA treatment. Transfection of control siRNA had no effect. Furthermore, we treated HCT116, HAB-68^mut^ and HAB-92^wt^ cells with a pharmacologic inhibitor. Sulindac is known to reduce *β*-catenin expression and its nuclear translocation, as well as to induce its proteasomal degradation, thereby modulating *β*-catenin target gene expression (Rice *et al*, 2003; Boon *et al*, 2004; Gardner *et al*, 2004; Han *et al*, 2008; Figure 1D). Here we probed the effect of sulindac on the *β*-catenin target gene *ABCB1*. ABCB1 expression levels in solvent-treated cells were dependent on *β*-catenin genotype, with reduced ABCB1 expression in HAB-92^wt^ cells harbouring only the wt allele of *β*-catenin. Sulindac treatment reduced ABCB1 mRNA expression in HCT116, HAB-68^mut^ and HAB-92^wt^ cells by seven-fold, five-fold and five-fold, respectively.

**Figure 2:**
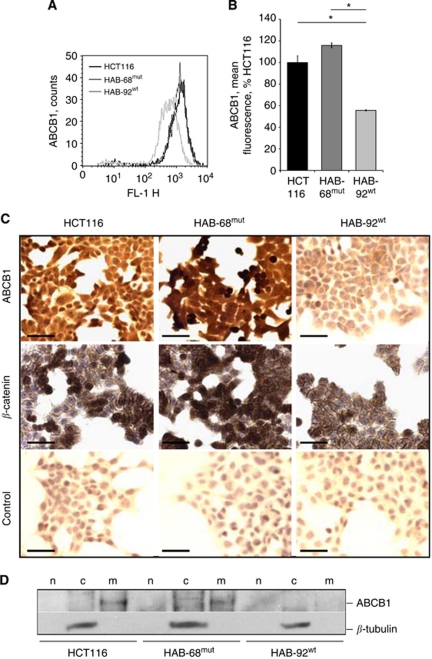
Gain-of-function *β*-catenin regulates ABCB1 protein expression in a dominant fashion. (**A**, **B**) ABCB1 protein expression analysis in HCT116, HAB-68^mut^ and HAB-92^wt^ cells. High ABCB1 levels were observed in the cell line HCT116 and its derivatives harbouring mut *β*-catenin, significantly lower ABCB1 expression in the derivatives with wt *β*-catenin, as determined by immuno flow cytometry. ^*^P<0.05. (**C**) Strong staining for ABCB1 was observed in cells harbouring mut *β*-catenin, HCT116 and HAB-68^mut^, compared with HAB-92^wt^ cells, as detected by immunocytochemistry; bar, 100 *μ*m. (**D**) Western blot analysis for ABCB1 in the nuclear, cytoplasmic and membranous fractions of HCT116, HAB-68^mut^ and HAB-92^wt^ cells. ABCB1 was found in the membrane fractions of HCT116 and HAB-68^mut^ cells, whereas membranous ABCB1 was not detected in HAB-92^wt^ cells. Abbreviations: c=cytoplasmic fraction; m=membranous fraction; n=nuclear fraction.

On the protein level, we found two-fold higher ABCB1 expression in cells harbouring mut *β*-catenin, HCT116 and HAB-68^mut^, compared with HAB-92^wt^ cells by immuno flow cytometry (Figure 2A and B). Immunocytochemistry confirmed these data: strong ABCB1 protein signals were observed in HCT116 and HAB-68^mut^ cells and much lower signals were observed in HAB-92^wt^ cells that express wt *β*-catenin exclusively (Figure 2C). Next, we analysed ABCB1 protein expression by western blotting, using isolated nuclear, cytoplasmic and membrane fractions of the cells (Figure 2D). Interestingly, we found ABCB1 in the membrane fractions of HCT116 and HAB-68^mut^ cells, whereas membranous ABCB1 was not detected in HAB-92^wt^ cells.

### ABCB1 promoter activity depends on *β*-catenin mutation status

**Figure 3:**
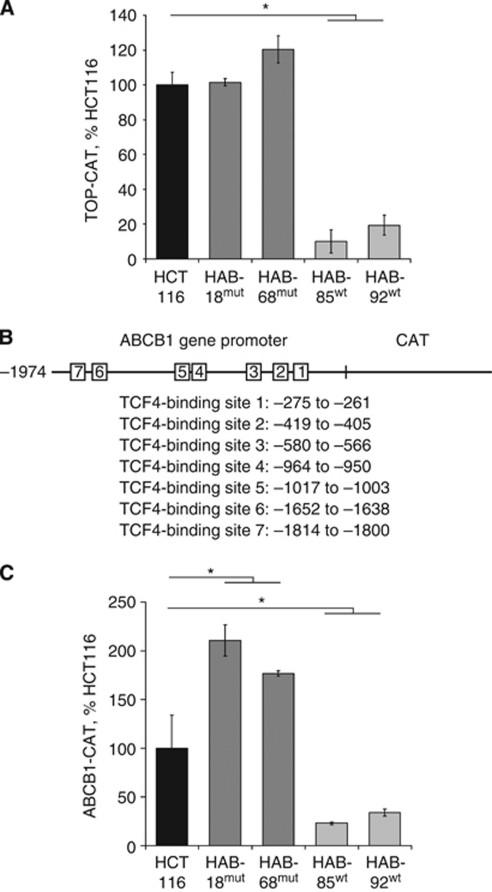
*ABCB1* gene promoter activity depends on *β*-catenin mutation status. (**A**, **C**) CAT-ELISA for TOP promoter activity (**A**) and for ABCB1 promoter activity (**C**) in HCT116, HAB-18^mut^, HAB-68^mut^, HAB-85^wt^ and HAB-92^wt^ cells. TOP promoter- (**A**) and ABCB1 promoter- (**C**) driven reporter gene expressions were strongly enhanced in cells harbouring gain-of-function *β*-catenin compared with the cell lines where the mut *β*-catenin allele was ablated. Transfections with the plasmid pFOP-CAT (**A**) and with the promoter-less plasmid pCAT3-basic (**C**) as well as transfections without DNA served as controls. The amount of CAT protein was normalised to the protein content of the respective lysate, expressed as pg CAT per mg protein and calculated as percentage of CAT reporter gene expression in HCT116 cells. Values are given as the average of triplicates. (**B**) Schematic drawing of the ABCB1 promoter, the TCF4-binding sites are marked. Nucleotide sequence and regulatory elements of the 5′-flanking region of the human *ABCB1* gene are based on Kohno *et al* (1990) and Yamada *et al* (2000). ^*^P<0.05.

Functional assays with the TCF4 reporter TOP-CAT confirmed that cells with the oncogenic allele of *β*-catenin had elevated levels of TCF4-mediated transactivation (Figure 3A). Substantially, up to 10-fold reduced signals were observed in the cell strains in which the mut allele was knocked out. To examine whether the *β*-catenin genotype determines the ABCB1 promoter activity, we searched the promoter for response elements for TCF4 proteins. Within the ABCB1 promoter fragment −1974 to +121 (kindly obtained from K Kohno), seven TCF4-binding sites were identified at −275 to −261, −419 to 405, −580 to −566, −964 to −950, −1017 to −1003, −1652 to 1638 and −1814 to −1800 (Kohno *et al*, 1990; Yamada *et al*, 2000) (Figure 3B). The ABCB1 promoter-driven CAT reporter gene construct was transiently transfected into HCT116 cells and knockout strains with gain or loss of function *β*-catenin. We observed an up to 10-fold higher reporter activity in cells with mut *β*-catenin, HCT116, HAB-18^mut^ and HAB-68^mut^, compared with HAB-85^wt^ and HAB-92^wt^ cells harbouring wt *β*-catenin exclusively (Figure 3C).

### *β*-Catenin mutation, subcellular localisation and ABCB1 expression in human colon carcinomas

**Figure 4:**
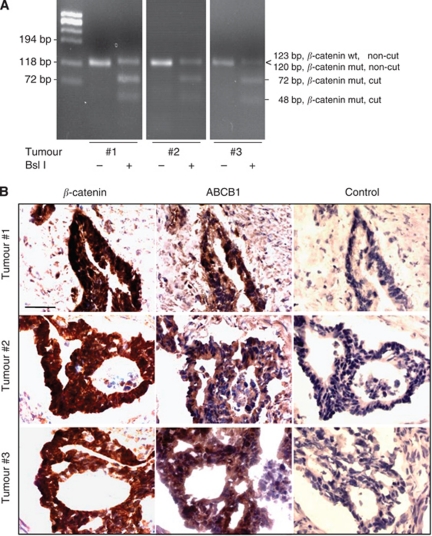
*β*-Catenin and ABCB1 expression in human colon carcinomas. (**A**) Thirty three primary colon carcinomas from untreated patients were analysed for *β*-catenin Δ45 mutation by RT–PCR-based restriction fragment length polymorphism (Stein *et al*, 2006). We identified three tumours to be heterozygous for the *β*-catenin Δ45 mutation. The mut *β*-catenin RT–PCR product is cut by Bsl I (fragments 72 and 48 bp), whereas the wt *β*-catenin RT–PCR product (123 bp) is not. (**B**) The nuclear localisation of *β*-catenin is clearly seen in these three tumours, together with high expression levels of ABCB1 protein. Subcellular localisation of *β*-catenin as well as ABCB1 protein expression was determined by immunohistochemistry in consecutive sections of these heterozygous tumours. Sections without primary antibodies served as controls; bars, 20 *μ*m.

We analysed tissue specimens from 33 colon cancer patients with adenocarcinomas of UICC stages I, II and III. These patients did not receive any pretreatment (for further characteristics: see Materials and Methods). Using a previously developed RT–PCR-based restriction fragment length polymorphism analysis, we characterised these specimens for their *β*-catenin mutation status (Stein *et al*, 2006). We identified three of these patient tumours that harbor the heterozygous in-frame deletion mutation of Δ45 in exon 3 of the *β*-catenin gene (Figure 4A). Consistent with the presence of this mutation, we observed nuclear localisation of *β*-catenin together with high ABCB1 levels in all three tumours that are heterozygous for mut *β*-catenin mutation (Figure 4B).

### *β*-Catenin mutation status and expression of ABCC1, ABCG2 and MVP

**Figure 5:**
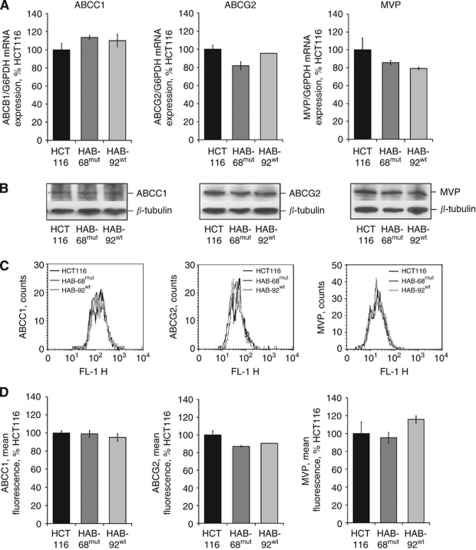
Expression of ABCC1, ABCG2 and MVP is independent of gain-of-function *β*-catenin. ABCC1, ABCG2 and MVP were not differentially expressed in cells with different *β*-catenin genotype. (**A**) ABCC1, ABCG2 and MVP mRNA expressions were determined by quantitative real-time RT–PCR; values represent the ratios of gene and G6PDH control mRNA (HCT116 cells: set 100%). Experiments were performed in duplicate and average values are shown. At the protein level, no significantly different expression levels of ABCC1, ABCG2 and MVP have been detected by western blot analysis (**B**) or by immuno flow cytometry (**C**, **D**).

We also analysed the expression of ABCC1, ABCG2 and MVP in HCT116, HAB-68^mut^ and HAB-92^wt^ cells. Expression of each of these chemoresistance-associated genes remained unchanged in the knockout sublines compared with HCT116, and was not dependent on the *β*-catenin genotype as demonstrated at the mRNA level by quantitative RT–PCR (Figure 5A), and at the protein level by western blot analyses (Figure 5B) as well as by immuno flow cytometry (Figure 5C and D).

### *β*-Catenin mutation status and *in vitro* sensitivity towards antitumour drugs

**Figure 6:**
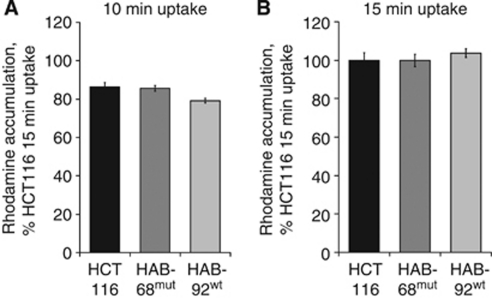
Rhodamine 123 accumulation assay. Functional impact of gain-of-function *β*-catenin was tested by incubating HCT116, HAB-68^mut^ and HAB-92^wt^ cells for 10 min (**A**) and 15 min (**B**) with rhodamine 123. Fluorescence intensity of the cells was measured after another 60 min in rhodamine 123-free medium. Despite their different *β*-catenin genotype and ABCB1 expression, no differences in accumulation of rhodamine 123 were measured.

To analyse whether drug accumulation is affected in cells with different *β*-catenin genotype and ABCB1 expression, we performed accumulation assays for rhodamine 123. However, rhodamine 123 accumulation was comparable in all the three cell lines, either after 10 min or 15 min of rhodamine 123 uptake (Figure 6A and B), despite different expression *β*-catenin genotype and ABCB1 levels.

**Figure 7:**
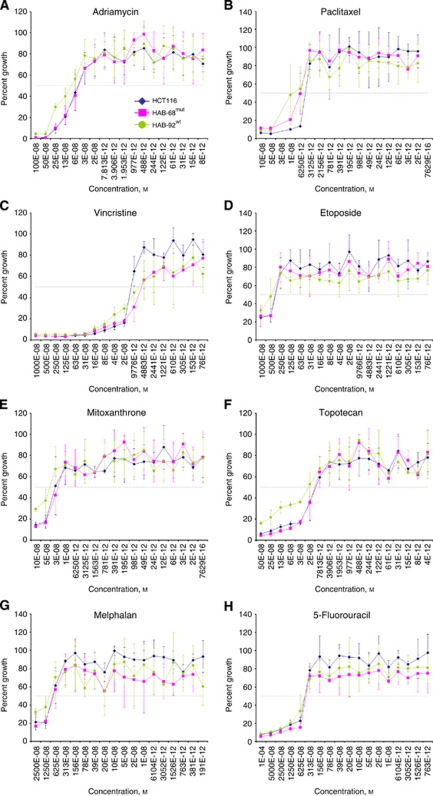
*β*-Catenin genotype did not alter chemosensitivity towards chemotherapeutic drugs. By high throughput screening, chemotherapeutic drugs were applied in 18 different concentrations to HCT116, HAB-68^mut^ and HAB-92^wt^ cells (for IC_50_ and for additional drugs see Table 1). Treatment responses of these cell lines towards the MDR-associated drugs adriamycin (**A**), paclitaxel (**B**), vincristine (**C**), etoposide (**D**), mitoxanthrone (**E**) and topotecan (**F**), as well as towards the chemotherapeutics melphalan (**G**) and 5-fluorouracil (**H**) are shown. Chemosensitivity of HCT116, HAB-68^mut^ and HAB-92^wt^ cells did not differ despite their differences in *β*-catenin genotype and ABCB1 expression levels. Cell survival is expressed as percent of control growth. Values are given as averages of triplicates.

As shown in Table 1 and Figure 7, the response of HCT116 and the *β*-catenin knockout cell lines to a mechanistically diverse library of anticancer drugs and prototype compounds did not reveal evidence of drug resistance mediated by *β*-catenin. A focus on known MDR substrates in this library does not indicate a consistent pattern of relative resistance based on the presence of oncogenic *β*-catenin. Indeed, the response of HCT116 and the derived knockout cell lines to the 24 compounds in this library were remarkably similar.

## Discussion

In this report we addressed the potential interplay between Wnt/*β*-catenin pathway activation and intrinsic response to chemotherapy, as activation of this pathway is observed in almost all colorectal cancers. We investigated this hypothesis using a frequently occuring mutation of *β*-catenin in the context of its MDR-associated target gene *ABCB1*. Here we report that mut *β*-catenin signalling regulates the expression of the *ABCB1* gene in a dominant fashion. We investigated the relevance of mut *β*-catenin with respect to ABCB1 expression levels to clinical cancer with surgical samples of primary colon carcinomas. We demonstrate that tumours heterozygous for mut *β*-catenin showed nuclear *β*-catenin together with overexpression of ABCB1. However, chemosensitivities towards MDR-related as well as non-related antitumour compounds measured by high throughput screening in tumour cell lines harbouring gain-of-function or wt *β*-catenin did not differ despite their differences in *β*-catenin genotype and ABCB1 expression levels.

We began our studies with HCT116 cells that are heterozygous for gain-of-function *β*-catenin (Chan *et al*, 2002; Kim *et al*, 2002). To address the role of gain-of-function *β*-catenin, knockout strains harbouring either the mut or the wt *β*-catenin allele exclusively, and cell clones with reconstituted heterozygous *β*-catenin genotype were analysed (Kim *et al*, 2002). We clearly demonstrated that cells with gain-of-function *β*-catenin, either intrinsically or by transfection, show higher ABCB1 levels than cells with wt *β*-catenin. Moreover, consistent with the *β*-catenin mutation status, we found higher ABCB1 promoter activity in cells harbouring gain-of-function *β*-catenin. These findings underline in particular that gain-of-function *β*-catenin acts in a dominant manner to control ABCB1 transcription. Thus, the dependence of ABCB1 expression on *β*-catenin genotype was clearly shown using this cell line model. Furthermore, intervening in *β*-catenin expression using siRNA acting on *β*-catenin led to ABCB1 expression knockdown. Treatment with sulindac reduced ABCB1 expression independent of the *β*-catenin genotype. Sulindac inhibits expression and nuclear accumulation of *β*-catenin in colorectal cancer cell lines, and induces proteasome-dependent degradation of *β*-catenin. Consequently, *β*-catenin target genes like Met, c-myc, cyclinD1 and S100A4 are downregulated following sulindac treatment (Rice *et al*, 2003; Boon *et al*, 2004; Gardner *et al*, 2004; Han *et al*, 2008; Stein *et al*, 2011). Thus, downregulation of ABCB1 by sulindac further supports the role of ABCB1 as *β*-catenin transcriptional target.

Our findings are in line with observations of Yamada *et al* (2000, 2003), who employed microarray technology in the colorectal cell line DLD1 and identified ABCB1 to be transcriptionally downregulated after inactivation of TCF4. The positive correlation of expression of *β*-catenin and ABCB1 was also shown in side-population colon cancer cells, however, ABCG2 expression was also found to be dependent on *β*-catenin levels (Chikazawa *et al*, 2010). The dependence of ABCB1 expression on *β*-catenin signalling was also reported for breast cancer cells (Bourguignon *et al*, 2009; Liu *et al*, 2010).

A large number of genes have been identified as *β*-catenin targets, including c-myc and cyclin D1, which are implicated in the development of colorectal cancer (Arber *et al*, 1996; He *et al*, 1998; Klaus and Birchmeier, 2008; Najdi *et al*, 2011). The importance of gain-of-function *β*-catenin has also been shown for *β*-catenin targets such as matrilysin, BMP4 and S100A4, thereby underlining the impact of activated *β*-catenin on tumourigenesis and metastasis (Kim *et al*, 2002; Ougolkov *et al*, 2002; Stein *et al*, 2006). Although *β*-catenin/TCF4-mediated ABCB1 expression represents only one mechanism within the complex transcriptional regulation of the *ABCB1* gene, our findings on the dominant action of gain-of-function *β*-catenin adds to the current knowledge on the role of ABCB1 in the biology of colorectal cancer.

We extended our *in vitro* analyses to archival samples of human primary cancer. We identified tumours that are heterozygous for this in-frame deletion mutation of Δ45 in exon 3 of the *β*-catenin gene. These tumours showed concomitant nuclear *β*-catenin and high ABCB1 expression. This finding, exploiting the defined Δ45 mutation as an example for gain-of-function *β*-catenin, provides a new link between high ABCB1 levels in human colon tumours and an activated Wnt pathway. Furthermore, increased ABCB1 expression also correlated with *β*-catenin in adenomatous polyps from patients with familial adenomatous polyposis (Yamada *et al*, 2000) as well as in side-population colon cancer cells (Chikazawa *et al*, 2010).

To assess the impact of gain-of-function *β*-catenin on ABCB1 and drug resistance and potential response to therapy in colon cancer cells we screened a mechanistically diverse library of anticancer drugs and prototype compounds. Using a high throughput screening approach designed to minimise the impact of drug sample handling on measured sensitivity, we found no evidence for dependence of cell response on *β*-catenin genotype. This was rather unexpected, particularly for MDR substrate drugs, in view of the clear evidence for upregulation of ABCB1 by oncogenic *β*-catenin. On the other hand, these results are consistent with the rhodamine efflux studies, which showed no difference between the cell lines. It may be that the extent of upregulation in these cell lines is not sufficient to confer functional drug resistance. It is also possible that expression of other genes in this HCT116-based cell line model, that is, *ABCC1*, *ABCG2* and *MVP*, contribute to the measured drug sensitivity phenotype. The simultaneous expression of MDR genes has already been described for HCT116 cells (Izquierdo *et al*, 1996; Stein *et al*, 1996, 1997a). We report here that the expression levels of ABCC1, ABCG2, and MVP were independent of the *β*-catenin genotype in HCT116 cells and the knockout sublines thereof. The simultaneous, unmodulated expression of the MDR-associated proteins ABCC1, ABCG2 and MVP may well contribute to the comparable chemosensitivities towards the 24 drugs tested.

In SW480 colon cancer cells, however, increased chemosensitivities towards paclitaxel and irinotecan were reported following *β*-catenin silencing by siRNA (Chikazawa *et al*, 2010). In breast cancer cells, knockdown of *β*-catenin by siRNA resulted in increased chemosensitivity towards doxorubicin and etoposide (Bourguignon *et al*, 2009); although these MDR-associated drugs are also transported by other ABC transporters than ABCB1, ABCB1 is highly overexpressed in this cell line when compared with other MDR-related genes (Stein *et al*, 1997b). For locally advanced breast cancer, no link between *β*-catenin and ABCB1 expression was found with respect to neoadjuvant chemotherapy (Shekhar *et al*, 2010).

For advanced and metastatic tumours, chemotherapy is frequently the only feasible treatment. However, the success of chemotherapy differs from patient to patient. Some patients show complete responses, others respond partially and/or transiently. Here we report the impact of gain-of-function *β*-catenin on intrinsic ABCB1 expression and *in vitro* chemotherapy response. We clearly demonstrate that the mutation status of *β*-catenin determines ABCB1 expression in a defined cell line model and in colon cancer specimens; however, no correlation of this finding with chemosensitivity towards 24 MDR-related and non-related antitumour compounds was detected. Further work is needed to evaluate the role of gain-of-function *β*-catenin with respect to additional anticancer compounds of potential use in treatment of colorectal cancer. Further studies will also be required to reveal the potential importance of an activated Wnt/*β*-catenin pathway for intrinsic ABCB1-mediated resistance in other tumour types. Likewise, additional insight is needed into factors important for expression of other ABC transporters, which may mediate drug resistance. In summary, although ABCB1 is validated as *β*-catenin target gene in a cell line model with defined *β*-catenin genotypes, the facile detection of *β*-catenin mutations using the diagnostic PCR procedure reported here is not sufficient for predicting therapy response or for individualisation of chemotherapy regimens for patients.

## Figures and Tables

**Table 1:** Chemosensitivity towards drugs, including MDR-associated compounds, as determined by high throughput screening in HCT116, HAB-68^mut^ and HAB-92^wt^ cells

**Drug**		**Cell line, IC_50_ (M)**		
**Name**	**Class (target)**	**HCT116**	**HAB-68^mut^**	**HAB-92^wt^**
Adriamycin^a^	Standard cytotoxic agent	5,90E-08	7,90E-08	1,06E-07
Paclitaxel^a^	Standard cytotoxic agent	3,45E-09	4,25E-09	7,10E-09
Vincristine^a^	Standard cytotoxic agent	1,20E-08	5,80E-09	7,10E-09
Etoposide (VP-16)^a^	Standard cytotoxic agent	3,60E-06	3,70E-06	4,70E-06
Mitoxanthrone^a^	Standard cytotoxic agent	2,15E-08	5,80E-08	6,85E-08
Topotecan^a^	Standard cytotoxic agent	1,05E-08	2,00E-08	4,85E-08
Melphalen^a^	Standard cytotoxic agent	7,60E-06	7,10E-06	9,60E-06
5-Fluorouracil^a^	Standard cytotoxic agent	4,40E-06	4,10E-06	4,50E-06
Gemcitabine	Standard cytotoxic agent	9,70E-10	8,30E-10	1,70E-09
5-Azacytidine	Epigenetic modulator	1,60E-06	3,70E-07	1,10E-06
PXD101	Epigenetic modulator	4,60E-07	3,30E-07	4,20E-07
Cyclopamine	Hedgehog pathway	8,60E-06	9,80E-06	1,10E-05
Bortezomid (Velcade)	Proteasome	4,50E-09	4,10E-09	6,20E-09
17-DMAG	Heat shock protein	7,20E-08	6,30E-08	1,50E-07
RHPS4	Telomerase	1,80E-05	1,80E-05	3,40E-05
Dasatinib	Kinases	3,10E-08	1,80E-08	1,30E-08
Erlotinib	Kinases	1,30E-04	1,30E-04	1,30E-04
Gefitinib (Iressa)	Kinases	1,60E-05	1,60E-05	1,70E-05
Imatinib (Gleevec)	Kinases	2,00E-05	2,00E-05	2,50E-05
Lapatinib	Kinases	1,50E-05	1,50E-05	2,00E-05
PV 1019	Kinases	1,70E-05	1,60E-05	1,70E-05
Sunitinib	Kinases	7,10E-06	5,90E-06	9,40E-06
Sorafenib	Kinases	5,10E-06	4,20E-06	5,50E-06
SU11274 (Sigma S9820)	Kinases	5,80E-06	3,70E-06	8,20E-06

By high throughput screening, 24 chemotherapeutic drugs were applied in 18 different concentrations to HCT116, HAB-68^mut^ and HAB-92^wt^ cells. Chemosensitivities of HCT116, HAB-68^mut^ and HAB-92^wt^ cells, expressed as IC_50_ values and given as averages of triplicates, did not differ significantly despite their differences in *β*-catenin genotype and ABCB1 expression levels, when comparing HAB-92^wt^ with HCT116 cells, or HAB-92^wt^ with HAB-68^mut^ cells.

a Concentration-dependent growth of HCT116, HAB-68^mut^ and HAB-92^wt^ cells, treated with the marked drugs, is illustrated in Figure 7.

## References

[bib1] Arber N, Hibshoosh H, Moss SF, Sutter T, Zhang Y, Begg M, Wang S, Weinstein IB, Holt PR (1996) Increased expression of cyclin D1 is an early event in multistage colorectal carcinogenesis. Gastroenterology 110: 669–674 8608874 10.1053/gast.1996.v110.pm8608874

[bib2] Bienz M, Clevers H (2000) Linking colorectal cancer to Wnt signaling. Cell 103: 311–320 11057903 10.1016/s0092-8674(00)00122-7

[bib3] Boon EM, Keller JJ, Wormhoudt TA, Giardiello FM, Offerhaus GJ, van der Neut R, Pals ST (2004) Sulindac targets nuclear beta-catenin accumulation and Wnt signalling in adenomas of patients with familial adenomatous polyposis and in human colorectal cancer cell lines. Br J Cancer 90: 224–229 14710233 10.1038/sj.bjc.6601505 PMC2395323

[bib4] Bourguignon LY, Xia W, Wong G (2009) Hyaluronan-mediated CD44 interaction with p300 and SIRT1 regulates beta-catenin signaling and NFkappaB-specific transcription activity leading to MDR1 and Bcl-xL gene expression and chemoresistance in breast tumor cells. J Biol Chem 284: 2657–2671 19047049 10.1074/jbc.M806708200 PMC2631959

[bib5] Chan TA, Wang Z, Dang LH, Vogelstein B, Kinzler KW (2002) Targeted inactivation of CTNNB1 reveals unexpected effects of beta-catenin mutation. Proc Natl Acad Sci USA 99: 8265–8270 12060769 10.1073/pnas.082240999 PMC123056

[bib6] Chikazawa N, Tanaka H, Tasaka T, Nakamura M, Tanaka M, Onishi H, Katano M (2010) Inhibition of Wnt signaling pathway decreases chemotherapy-resistant side-population colon cancer cells. Anticancer Res 30: 2041–2048 20651349

[bib7] Cordon-Cardo C, O’Brien JP, Boccia J, Casals D, Bertino JR, Melamed MR (1990) Expression of the multidrug resistance gene product (P-glycoprotein) in human normal and tumor tissues. J Histochem Cytochem 38: 1277–1287 1974900 10.1177/38.9.1974900

[bib8] Fearon ER, Vogelstein B (1990) A genetic model for colorectal tumorigenesis. Cell 61: 759–767 2188735 10.1016/0092-8674(90)90186-i

[bib9] Gardner SH, Hawcroft G, Hull MA (2004) Effect of nonsteroidal anti-inflammatory drugs on beta-catenin protein levels and catenin-related transcription in human colorectal cancer cells. Br J Cancer 91: 153–163 15188006 10.1038/sj.bjc.6601901 PMC2364748

[bib10] Gillet JP, Gottesman MM (2011) Advances in the molecular detection of ABC transporters involved in multidrug resistance in cancer. Curr Pharm Biotechnol 12: 686–692 21118086 10.2174/138920111795163931 PMC3188423

[bib11] Gottesman MM, Fojo T, Bates SE (2002) Multidrug resistance in cancer: role of ATP-dependent transporters. Nature Rev 2: 48–58 10.1038/nrc706 11902585

[bib12] Han A, Song Z, Tong C, Hu D, Bi X, Augenlicht LH, Yang W (2008) Sulindac suppresses beta-catenin expression in human cancer cells. Eur J Pharmacol 583: 26–31 18291362 10.1016/j.ejphar.2007.12.034 PMC2350231

[bib13] He TC, Sparks AB, Rago C, Hermeking H, Zawel L, da Costa LT, Morin PJ, Vogelstein B, Kinzler KW (1998) Identification of c-MYC as a target of the APC pathway. Science 281: 1509–1512 9727977 10.1126/science.281.5382.1509

[bib14] Ho GT, Moodie FM, Satsangi J (2003) Multidrug resistance 1 gene (P-glycoprotein 170): an important determinant in gastrointestinal disease? Gut 52: 759–766 12692067 10.1136/gut.52.5.759 PMC1773632

[bib15] Ilyas M, Tomlinson IP, Rowan A, Pignatelli M, Bodmer WF (1997) Beta-catenin mutations in cell lines established from human colorectal cancers. Proc Natl Acad Sci USA 94: 10330–10334 9294210 10.1073/pnas.94.19.10330 PMC23362

[bib16] Izquierdo MA, Shoemaker RH, Flens MJ, Scheffer GL, Wu L, Prather TR, Scheper RJ (1996) Overlapping phenotypes of multidrug resistance among panels of human cancer-cell lines. Int J Cancer 65: 230–237 8567122 10.1002/(SICI)1097-0215(19960117)65:2<230::AID-IJC17>3.0.CO;2-H

[bib17] Kim IJ, Kang HC, Park JH, Shin Y, Ku JL, Lim SB, Park SY, Jung SY, Kim HK, Park JG (2003) Development and applications of a beta-catenin oligonucleotide microarray: beta-catenin mutations are dominantly found in the proximal colon cancers with microsatellite instability. Clin Cancer Res 9: 2920–2925 12912937

[bib18] Kim JS, Crooks H, Dracheva T, Nishanian TG, Singh B, Jen J, Waldman T (2002) Oncogenic beta-catenin is required for bone morphogenetic protein 4 expression in human cancer cells. Cancer Res 62: 2744–2748 12019147

[bib19] Klaus A, Birchmeier W (2008) Wnt signalling and its impact on development and cancer. Nat Rev Cancer 8: 387–398 18432252 10.1038/nrc2389

[bib20] Kohno K, Sato S, Uchiumi T, Takano H, Kato S, Kuwano M (1990) Tissue-specific enhancer of the human multidrug-resistance (MDR1) gene. J Biol Chem 265: 19690–19696 1978833

[bib21] Litman T, Druley TE, Stein WD, Bates SE (2001) From MDR to MXR: new understanding of multidrug resistance systems, their properties and clinical significance. Cell Mol Life Sci 58: 931–959 11497241 10.1007/PL00000912 PMC11337370

[bib22] Liu YY, Gupta V, Patwardhan GA, Bhinge K, Zhao Y, Bao J, Mehendale H, Cabot MC, Li YT, Jazwinski SM (2010) Glucosylceramide synthase upregulates MDR1 expression in the regulation of cancer drug resistance through cSrc and beta-catenin signaling. Mol Cancer 9: 145 20540746 10.1186/1476-4598-9-145 PMC2903501

[bib23] Morin PJ, Sparks AB, Korinek V, Barker N, Clevers H, Vogelstein B, Kinzler KW (1997) Activation of beta-catenin-Tcf signaling in colon cancer by mutations in beta-catenin or APC. Science 275: 1787–1790 9065402 10.1126/science.275.5307.1787

[bib24] Najdi R, Holcombe RF, Waterman ML (2011) Wnt signaling and colon carcinogenesis: beyond APC. J Carcinog 10: 5 21483657 10.4103/1477-3163.78111 PMC3072659

[bib25] Orford K, Crockett C, Jensen JP, Weissman AM, Byers SW (1997) Serine phosphorylation-regulated ubiquitination and degradation of beta-catenin. J Biol Chem 272: 24735–24738 9312064 10.1074/jbc.272.40.24735

[bib26] Ougolkov AV, Yamashita K, Mai M, Minamoto T (2002) Oncogenic beta-catenin and MMP-7 (matrilysin) cosegregate in late-stage clinical colon cancer. Gastroenterology 122: 60–71 11781281 10.1053/gast.2002.30306

[bib27] Polakis P (1999) The oncogenic activation of beta-catenin. Curr Opin Genet Dev 9: 15–21 10072352 10.1016/s0959-437x(99)80003-3

[bib28] Polakis P (2000) Wnt signaling and cancer. Genes Dev 14: 1837–1851 10921899

[bib29] Provost E, Yamamoto Y, Lizardi I, Stern J, D’Aquila TG, Gaynor RB, Rimm DL (2003) Functional correlates of mutations in beta-catenin exon 3 phosphorylation sites. J Biol Chem 278: 31781–31789 12799363 10.1074/jbc.M304953200

[bib30] Rice PL, Kelloff J, Sullivan H, Driggers LJ, Beard KS, Kuwada S, Piazza G, Ahnen DJ (2003) Sulindac metabolites induce caspase- and proteasome-dependent degradation of beta-catenin protein in human colon cancer cells. Mol Cancer Ther 2: 885–892 14555707

[bib31] Riordan JR, Ling V (1979) Purification of P-glycoprotein from plasma membrane vesicles of Chinese hamster ovary cell mutants with reduced colchicine permeability. J Biol Chem 254: 12701–12705 500733

[bib32] Shekhar MP, Biernat LA, Pernick N, Tait L, Abrams J, Visscher DW (2010) Utility of DNA postreplication repair protein Rad6B in neoadjuvant chemotherapy response. Med Oncol 27: 466–473 19466589 10.1007/s12032-009-9235-7

[bib33] Stein U, Arlt F, Smith J, Sack U, Herrmann P, Walther W, Lemm M, Fichtner I, Shoemaker RH, Schlag PM (2011) Intervening in *β*-catenin signaling by sulindac inhibits S100A4-dependent colon cancer metastasis. Neoplasia 13: 131–144 21403839 10.1593/neo.101172 PMC3033592

[bib34] Stein U, Arlt F, Walther W, Smith J, Waldman T, Harris ED, Mertins SD, Heizmann CW, Allard D, Birchmeier W, Schlag PM, Shoemaker RH (2006) The metastasis-associated gene S100A4 is a novel target of beta-catenin/T-cell factor signaling in colon cancer. Gastroenterology 131: 1486–1500 17101323 10.1053/j.gastro.2006.08.041

[bib35] Stein U, Jürchott K, Schläfke M, Hohenberger P (2002) Expression of multidrug resistance genes MVP, MDR1, and MRP1 determined sequentially before, during, and after hyperthermic isolated limb perfusion of soft tissue sarcoma and melanoma patients. J Clin Oncol 20: 3282–3292 12149303 10.1200/JCO.2002.01.003

[bib36] Stein U, Walther W (2006) Current opinion: reversal of ABC transporter-mediated multidrug resistance in cancer: a realistic option? Am J Cancer 5: 285–297

[bib37] Stein U, Walther W, Arlt F, Schwabe H, Smith J, Fichtner I, Birchmeier W, Schlag PM (2009) MACC1, a newly identified key regulator of HGF-MET signaling, predicts colon cancer metastasis. Nat Med 15: 59–67 19098908 10.1038/nm.1889

[bib38] Stein U, Walther W, Laurencot CM, Scheffer GL, Scheper RJ, Shoemaker RH (1997a) Tumor necrosis factor-alpha and expression of the multidrug resistance-associated genes LRP and MRP. J Natl Cancer Inst 89: 807–813 9182980 10.1093/jnci/89.11.807

[bib39] Stein U, Walther W, Lemm M, Naundorf H, Fichtner I (1997b) Development and characterisation of novel human multidrug resistant mammary carcinoma lines *in vitro* and *in vivo*. Int J Cancer 72: 885–891 9311609 10.1002/(sici)1097-0215(19970904)72:5<885::aid-ijc28>3.0.co;2-6

[bib40] Stein U, Walther W, Shoemaker RH (1996) Reversal of multidrug resistance by transduction of cytokine genes into human colon carcinoma cells. J Natl Cancer Inst 88: 1383–1392 8827016 10.1093/jnci/88.19.1383

[bib41] Szakacs G, Paterson JK, Ludwig JA, Booth-Genthe C, Gottesman MM (2006) Targeting multidrug resistance in cancer. Nat Rev Drug Discov 5: 219–234 16518375 10.1038/nrd1984

[bib42] Thiebaut F, Tsuruo T, Hamada H, Gottesman MM, Pastan I, Willingham MC (1987) Cellular localization of the multidrug-resistance gene product P-glycoprotein in normal human tissues. Proc Natl Acad Sci USA 84: 7735–7738 2444983 10.1073/pnas.84.21.7735 PMC299375

[bib43] Tiwari AK, Sodani K, Dai CL, Ashby Jr CR, Chen ZS (2011) Revisiting the ABCs of multidrug resistance in cancer chemotherapy. Curr Pharm Biotechnol 12: 570–594 21118094 10.2174/138920111795164048

[bib44] Tusnady GE, Sarkadi B, Simon I, Varadi A (2006) Membrane topology of human ABC proteins. FEBS Lett 580: 1017–1022 16337630 10.1016/j.febslet.2005.11.040

[bib45] Ueda K, Cardarelli C, Gottesman MM, Pastan I (1987) Expression of a full-length cDNA for the human MDR1 gene confers resistance to colchicine, doxorubicin, and vinblastine. Proc Natl Acad Sci USA 84: 3004–3008 3472246 10.1073/pnas.84.9.3004 PMC304789

[bib46] van de Wetering M, Sancho E, Verweij C, de Lau W, Oving I, Hurlstone A, van der Horn K, Batlle E, Coudreuse D, Haramis AP, Tjon-Pon-Fong M, Moerer P, van den Born M, Soete G, Pals S, Eilers M, Medema R, Clevers H (2002) The beta-catenin/TCF-4 complex imposes a crypt progenitor phenotype on colorectal cancer cells. Cell 111: 241–250 12408868 10.1016/s0092-8674(02)01014-0

[bib47] Vogelstein B, Kinzler KW (2004) Cancer genes and the pathways they control. Nat Med 10: 789–799 15286780 10.1038/nm1087

[bib48] Weinstein RS, Jakate SM, Dominguez JM, Lebovitz MD, Koukoulis GK, Kuszak JR, Klusens LF, Grogan TM, Saclarides TJ, Roninson IB, Coon JS (1991) Relationship of the expression of the multidrug resistance gene product (P-glycoprotein) in human colon carcinoma to local tumor aggressiveness and lymph node metastasis. Cancer Res 51: 2720–2726 1673639

[bib49] Wong SC, Lo ES, Lee KC, Chan JK, Hsiao WL (2004) Prognostic and diagnostic significance of beta-catenin nuclear immunostaining in colorectal cancer. Clin Cancer Res 10: 1401–1408 14977843 10.1158/1078-0432.ccr-0157-03

[bib50] Wu L, Smythe AM, Stinson SF, Mullendore LA, Monks A, Scudiero DA, Paull KD, Koutsoukos AD, Rubinstein LV, Boyd MR, Shoemaker RH (1992) Multidrug-resistant phenotype of disease-oriented panels of human tumor cell lines used for anticancer drug screening. Cancer Res 52: 3029–3034 1350507

[bib51] Yamada T, Mori Y, Hayashi R, Takada M, Ino Y, Naishiro Y, Kondo T, Hirohashi S (2003) Suppression of intestinal polyposis in Mdr1-deficient ApcMin/+ mice. Cancer Res 63: 895–901 12615699

[bib52] Yamada T, Takaoka AS, Naishiro Y, Hayashi R, Maruyama K, Maesawa C, Ochiai A, Hirohashi S (2000) Transactivation of the multidrug resistance 1 gene by T-cell factor 4/beta-catenin complex in early colorectal carcinogenesis. Cancer Res 60: 4761–4766 10987283

